# Rapid Detection of *Candida tropicalis* in Clinical Samples From Different Sources Using RPA-LFS

**DOI:** 10.3389/fcimb.2022.898186

**Published:** 2022-07-07

**Authors:** Lei Wang, Aiguo Xu, Ping Zhou, Mengdi Zhao, Chenglai Xu, Yan Wang, Kun Wang, Fang Wang, Yongchang Miao, Weiguo Zhao, Xuzhu Gao

**Affiliations:** ^1^ Department of Central Laboratory, Lianyungang Hospital Affiliated to Jiangsu University (Cancer Hospital of Lianyungang), Lianyungang, China; ^2^ School of Biotechnology, Jiangsu University of Science and Technology, Zhenjiang, China; ^3^ Department of Materials Science and Engineering, Suzhou University of Science and Technology, Suzhou, China

**Keywords:** *C. tropicalis*, recombinase polymerase amplification, lateral flow strip, *ITS2* gene, Cryptococcaceae

## Abstract

*Candida tropicalis* is one of the few Candida species besides *Candida albicans* that is able to produce true hyphae. At present, the commonly used clinical methods for the identification of this organism are traditional fungal culture, CTB staining, and color development. Polymerase chain reaction (PCR) and real-time quantitative PCR (qPCR) are also used to identify this fungus. Since the course of *C*. *tropicalis* infection progresses rapidly, there is an urgent need for rapid, sensitive, real-time field assays to meet the needs of clinical diagnosis. Recombinase polymerase amplification (RPA) combined with lateral flow strip (LFS) can rapidly amplify and visualize target genes within 20 min, and by pre-processing samples from different sources, the entire process can be controlled within 30 min. In this study, RPA-LFS was used to amplify the internal transcribed spacer-2 (*ITS2*) gene of *C*. *tropicalis*, and primer-probe design was optimized by introducing base mismatches to obtain a specific and sensitive primer-probe combination for clinical sample detection. LFS assay for 37 common clinical pathogens was performed, sensitivity and specificity of the detection system was determined, reaction temperature and time were optimized, and 191 actual clinical samples collected from different sources were tested to evaluate the detection performance of the established RPA-LFS system to provide a reliable molecular diagnostic method for the detection of *C*. *tropicalis*, the results show that the RPA-LFS system can specifically detect *C. tropicalis* without cross-reacting with other fungi or bacterial, with a sensitivity of 9.94 CFU/µL, without interference from genomic DNA of other species, at an optimal reaction temperature of 39°C, and the whole reaction process can be controlled within 20 min, and to meet the clinical need for rapid, sensitive, real-time, and portable field testing.

## Introduction

*C. tropicalis* is one of the few Candida species besides *C. albicans* that is able to produce true hyphae (Zuza-Alves et al., 2017). The overgrowth of yeast caused by pseudofilamentous yeast is also considered Candida, which can invade the skin, mucous membranes, and internal organs, manifesting as acute, subacute, or chronic inflammation, mostly secondary infection. There are many types of pseudomycetes, but only a few can cause disease in people; *C*. *tropicalis* is the second most common pathogenic fungi after *Candida albicans*. Other pathogenic Candida include *Candida parapsilosis*, *Candida dubliniensis*, *Candida krusei*, and *Candida carpophila* (Khodadadi et al., 2017).

The traditional fungal culture method is the gold standard for the diagnosis of fungal infectious diseases and can detect *C*. *tropicalis* infection from various specimens, such as sputum, urine, stool, blood, various secretions, and chest and abdominal fluid (Yong et al., 2008). In addition, the colorimetric method has been used clinically to distinguish *C*. *tropicalis* from many kinds of *Candida*, but, similar to the traditional fungal culture method, this method is time-consuming. Detection of *C*. *tropicalis* is easily confused with *Candida glabrata* in some colorimetric methods; *C. tropicalis* is not always accurately detected rapidly. Therefore, it is important to establish a rapid and effective method for detecting *C*. *tropicalis* (Baixench et al., 2006; Zhao et al., 2016).

With the continuous development of molecular diagnostic techniques, PCR (García-Salazar et al., 2022), qPCR (Makino et al., 2010), and LAMP-based nucleic acid detection are also increasingly used in the detection of *C*. *tropicalis* (Trabasso et al., 2015). In particular, isothermal amplification is gaining more attention in on-site testing because it is not restricted by instruments and laboratory scenarios. Recombinase polymerase amplification (RPA) is a recently emerged thermostatic amplification technology that combines the advantages of the above methods and makes up for the shortcomings to meet the diagnostic needs of rapid, on-site, sensitive, and portable assays (Frimpong et al., 2019). RPA uses a recombinase to open the double-strand and bind the primer to the target fragment, and the polymerase Bsu, which has chain-switching activity, to recognize the 3’ end of the primer for stable amplification (Piepenburg et al., 2006; Cossio et al., 2021).

The amplification products of RPA can be detected by gel electrophoresis (Hu et al., 2019), fluorescence detector (Liu et al., 2019), and LFS (Wu et al., 2017). However, the sensitivity of gel electrophoresis and fluorescence detection is limited outside the laboratory. By contrast, LFS is suitable for simple assays in which the results can be analyzed visually without the need for complex thermal cycling equipment and trained personnel. Visualization depends on the presence of a fluorescein isothiocyanate (FITC) tag and a C3 blocking site at the 5’ and 3’ ends of the probe, respectively. In the middle of the probe, a base is also substituted with tetrahydrofuran (THF). Binding of the probe to the amplified strand is initiated by cleavage of endonuclease IV (Nfo), which releases the 3’ end of the probe for extension. Using biotin-labeled reverse primers, the 5’ and 3’ ends of the amplification product are labeled with FITC and biotin, respectively. Anti-FITC antibodies labeled with gold nanoparticles (AuNP-labeled) on the conjugate pad of the LFS can bind the amplification product, which is subsequently captured and aggregated by a detection line coated with streptavidin, as indicated by a red positive signal. The RPA-LFS assay is useful as a diagnostic for various infectious diseases when the RPA-LFS assay can achieve fast response times and good accuracy when used as a diagnostic test for various infectious diseases (Rosser et al., 2015; Dai et al., 2019).

Currently, RPA-LFS has been increasingly used in the medical testing of various pathogenic microorganisms. The pathogenic microorganisms that have been reported in our laboratory to establish assays based on RPA-LFS technology include *Pseudomonas aeruginosa* (Yang et al., 2021), *Cryptococcus neoformans* (Wang et al., 2022), and *Candida albicans* (Wang et al., 2021). In this study, a detection technique was established using RPA-LFS technology for the rapid diagnosis of infections caused by *C*. *tropicalis*. With the establishment of a rapid fungal detection method, early detection results can guide the clinic to undertake appropriate treatment promptly, improving patient diagnosis and treatment.

## Materials and Methods

### Ethics Statement

This study was approved by the Medical Ethics Committee of the Second People’s Hospital of Lianyungang City (permit number: 2020013). The clinical strains were collected from 2020 to 2021. All samples were obtained with written consent.

### Strain Acquisition

*C. tropicalis* ATCC 20962/201380/1369/66029 was purchased from Shanghai Covey Chemical Technology Co., Ltd. Fifteen strains of *C*. *tropicalis* were isolated from clinical samples, and 37 other common pathogens were provided by our laboratory to verify the specificity of the RPA-LFS method based on the *ITS2* gene, including *C. albicans* ATCC 10231, *Candida parapsilosis*, *Candida dubliniensis*, *Candida krusei*, *Candida carpophila*, *Aspergillus fumigatus*, *Cryptococcus neoformans* ATCC 14116, *Enterococcus faecium*, *Escherichia coli* O157, *Mycobacterium tuberculosis* H37Ra, *Pseudomonas aeruginosa*, *Staphylococcus aureus*, *Staphylococcus capitis*, *Staphylococcus epidermidis*, *Staphylococcus haemolyticus*, *Staphylococcus hominis*, *Staphylococcus saprophytics*, *Staphylococcus wameri*, *Stenotrophomonas maltophilia*, *Streptococcus pneumonia*, *Viridans streptococci*, *Klebsiella pneumoniae*, *Haemophilus influenzae* ATCC 49247, *Acinetobacter baumannii* ATCC 19606, *Candida auris*, *Candida glabrata*, *Candida metapsilosis*, *Candida orthopsilosis*, *Cryptococcus gattii*, *Enterobacter cloacae*, *Listeria monocytogenes*, *Neisseria meningitidis*, *Acinetobacter calcoaceticus*, *Acinetobacter lwoffi*, *Acinetobacter haemolytius*, *Acinetobacter junii*, and *Acinetobacter johnsonii*. A total of 191 (blood 93, sputum 45, urine 36, and other samples 17) clinical samples suspected of fungal infection were collected and pre-processed by the microbiology team of our laboratory.

### DNA Extraction

All bacterial strains were incubated at 100°C for 10 min before being used as templates. If not specified, 1 μL of 10^5^ CFU/mL of heat-treated culture was used as the template. For *Candida tropicalis* and other fungi, usually weighing 0.1-0.5 g of wet fungal hyphae, spores, ascospores or precipitate obtained by centrifugation of 0.1-3 mL of liquid clinical specimens, transferred to plastic mortar, which requires pre-purification of the fungus from the environment (e.g. DNA extraction from blood pathogenic fungi requires removal of components such as red blood cells first), followed by genomic DNA extraction according to the instructions of the GeneJET Genomic DNA Purification Kit (Tiangen Biotechnology Co., Ltd., Beijing, China). Extracted genomic DNA was quantified using a Qubit 4 fluorometer (Thermo Fisher Scientific) according to the manufacturer’s instructions.

### Primer and Probe Design and Screening

Two pairs of RPA primers based on the *ITS2* gene were designed using Primer Premier 5.0 software (Premier Biosoft International, CA, USA). For the primers, after entering the sequence of the specific target region, the parameters were set as follows: product size was set to 100–300 bp, primer size was set to 30–35 bp, complementary pairing of no more than three consecutive bases at the 3′ end, maximum hairpin score was set to 9, maximum primer-dimer score was set to 9, and maximum poly-X was set to 5. The primers and probes were designed using Primer Premier 5.0 software on the NCBI website (https://www.ncbi.nlm.nih.gov/tools/primer-blast) on Primer-BLAST to confirm species specificity of the sequences. The forward primer 5, extended backward by 16 bp, was evaluated for probe and reverse primer performance using Primer Premier 5 software to limit the formation of dimers and hairpin structures between the probe and reverse primer, with a probe size of 46–51 bp, GC content of 20–80%, and Tm of 57–80°C. The 5’ end of the probe was labeled with FITC, the 3’ end was closed with a C3 spacer, the bases in the middle of the probe were replaced with tetrahydrofuran (THF) with at least 30 bp before the THF site and at least 15 bp afterwards, and the 5’ end of the reverse primer was labeled with biotin.

### RPA Reaction

RPA reactions were performed using the TwistAmp^®^ Liquid DNA Amplification Kit (TwistDx Inc., Maidenhead, UK) according to the manufacturer’s instructions. The 50 µL reaction system was prepared as follows: 25 µL of 2× reaction buffer, 5 µL of 10× Basic e-mix, 2.5 µL of 20× core mix, 2.4 µL of 10 µM forward primer, 2.4 µL of 10 µM reverse primer, and 9.2 µL of distilled water; 2.5 μL of 280 mM magnesium acetate and 1 μL of template were added to the lid of the reaction tube. After a short centrifugation, the reaction mixture was incubated at 37°C for 30 min. The RPA amplification products were purified using the PCR Cleanup Kit (Meiji Biotechnology, Shanghai, China) and electrophoresed on a 2% agarose gel.

### RPA-LFS

RPA reactions were performed according to the manufacturer’s instructions of the Twist Amp^®^ DNA amplification nfo kit (TwistDx Ltd., Maidenhead, UK). Each 25 μL reaction mixture contained 1.05 μL of each primer (10 μM), 0.3 μL of probe (10 μM), 1.0 μL of template, and other standard reaction components. Primers and probes were synthesized by Anhui General Biotechnology Co., Ltd. To initiate the reaction, 1.25 μL of magnesium acetate (280 mM) was added, and the reaction mixture was incubated for 30 min at 37°C. Then, 5 μL of the amplification product was spotted on the LFS (Usta Biotechnology Co., Ltd., Hangzhou, China). The LFS consisted of a sample pad, a gold-labeled antibody pad (soaked with mouse-derived AuNP-labeled anti-FITC antibody), a test line (coated with streptavidin), a control line (coated with anti-mouse antibody), and an absorption pad, arranged by the solvent migration pathway. The RPA amplification product is added to the sample pad of the LFS and the LFS was inserted into 100 μL of solvent for approximately 2 min until the test and control lines were visually inspected.

### qPCR

Primers and probes are listed in **Table 1**. Specific primers and probes targeting the *ITS2* gene of *C*. *tropicalis* (Yan et al., 2012) were used for qPCR assays. The qPCR reaction mixture consisted of 12.5 μL MonAmpTM Taqman qPCR mixture (Tiangen Biotechnology Co., Ltd., Beijing, China), 0.5 μM forward and reverse primers, 0.2 μM probe, 1 μL genomic DNA, and distilled water to 25 μL. The cycling program was 95°C for 10 min, followed by 40 cycles of 95°C for 15 s and 55°C for 60 s on a Roche LightCycler 480 qPCR machine.

**Table 1:** Primers and probes for *C. tropicalis*.

Primers/Probes	Primer Sequences	Size (bp)	Reaction name
*ITS2*-F1	TTATTATTACAATAGTCAAAACTTTCAACAACGGATC	37	RPA
*ITS2*-R1	GAATATCTGCAATTCATATTACGTATCGCATTT	33	
*ITS2*-F2	ATTATTACAATAGTCAAAACTTTCAACAACGGA	33	
*ITS2*-R2	ATTCACGAATATCTGCAATTCATATTACGTATC	33	
*ITS2*-P	FITC-TTATTATTACAGTAGTCAACACTTTCAGCAACGGATC[THF]CTTGATTCTCGCATC-C3 spacer、	52	RPA-LFS
*ITS2*-R1B	Biotin-GAACATCTTCACTTCATATTACGTATCGCATTT	33	
*ITS2*-F3	TTTTATTGAACAAATTTCTTTGGTGGCGGGAGC	33	
*ITS2*-F4	ATTGAACAAATTTCTTTGGTGGCGGGAGCAA	31	
*ITS2*-F5	TGAACAAATTTCTTTGGTGGCGGGAGCAATC	31	
*ITS2*-F6	AACAAATTTCTTTGGTGGCGGGAGCAATCCT	31	
*ITS2*-F7	CAAATTTCTTTGGTGGCGGGAGCAATCCTAC	31	
F	GCGTCATTTCTCCCTCAAACC	21	qPCR (Yan et al., 2012)
R	TAAGTTTCCACGTTAAATTCTTTCAAAC	28	
P	VIC d (AACCTAGCGTATTGCTCAACACCAAACCCG) BHQ1	30	

F, forward primer; R, reverse primer; P, probe.

## Results

### Primer Validation Screening Strategy

**Figure 1:**
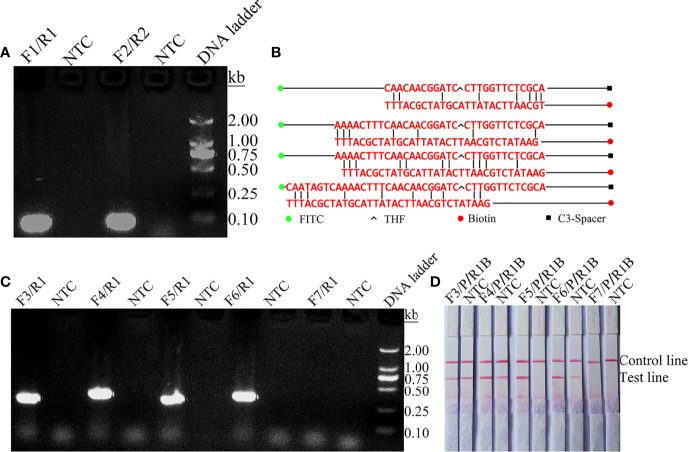
Screening of primer-probe combinations. **(A)** RPA results for two different primer sets of the *ITS2* gene. Primer sets are indicated at the top of each lane. No-template control (NTC) was used in the reactions. All reactions were performed at 37°C for 30 min. Images are representative of three independent experiments. **(B)** Pairwise analysis and sequence modification of the primer-probe sets used to detect the *ITS2* gene using Primer Premier 5 software. The associated DNA base substitutions of the probes and primers. The DNA strands are shown as horizontal lines and the matching bases are indicated by vertical lines. Molecular markers are listed under Figure **(B)**. **(C)** Agarose gel showing primers for RPA amplification using *C. tropicalis* genomic DNA as a template. The names of the primer pairs are indicated at the top of each lane. NTC indicates the no-template control for the respective primer pair. The band size of the DNA ladder is shown on the right. **(D)** Validity of primer-probe sets for the RPA-LFS assay. The name of each primer set is shown at the top of each lane, and NTC indicates the no template control for the respective primer pair. The positions of the test and control lines are shown on the right. All reactions were performed at 37°C for 30 min. Images are representative of three independent experiments.

The *ITS2* gene was selected from the *C*. *tropicalis* genome as a target for RPA-LFS detection. A primer search of the sequence of the *ITS2* gene using NCBI Primer-BLAST yielded two potential primer pairs (**Table 1**). These primers were initially screened by amplification of target gene fragments and template-free control. The amplification products were electrophoresed on an agarose gel to compare the amplification performance of the target gene with the formation of primer-dimers in the no-template control. Primer pairs that showed the best amplification performance and no primer dimer formation were selected (**Figure 1A**). The candidate probe was obtained by extending the 3’ end of the forward primer F2 by 16 bp. All possible cross-dimers generated by this probe and the reverse primer were predicted, and the bases were subsequently modified until no dimer could be formed (**Figure 1B**). Finally, for the five forward primers designed, screened, and tested upstream of the probe, the gel electrophoresis results showed that F3/R1, F4/R1, F5/R1, and F6/R1 were all able to effectively amplify the target gene fragment and that the negative control without added template did not amplify (**Figure 1C**). The RPA-LFS results showed that only F5/P/R1B met the requirements of the assay and was able to effectively amplify the *ITS2* gene fragment, and the negative control without added template was not amplified (**Figure 1D**). Therefore, F5/P/R1B was used in subsequent experiments.

### Optimization of RPA-LFS Reaction Conditions

**Figure 2:**
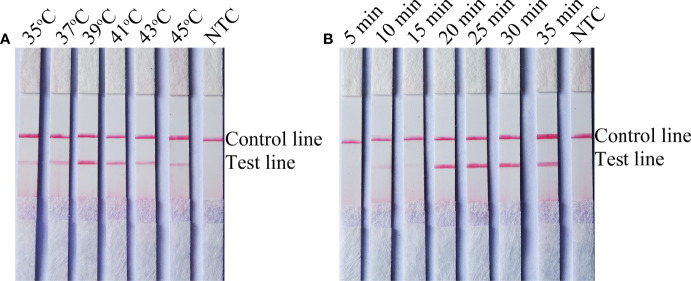
Screening of optimal temperature and time parameters for the *C. tropicalis* RPA-LFS system. **(A)** Performance of *C. tropicalis* RPA-LFS at temperatures ranging from 35°C to 45°C. **(B)** Performance of *C. tropicalis* RPA-LFS at times ranging from 5 min to 35 min. NTC lane is a no-template control.

The temperature parameters were set to 35–45˚C for 30 min. As shown in **Figure 2A**, the signal on the test line appears on each temperature gradient; the line is more obvious at 39°C. Time parameters were set from 5 min to 35 min. As shown in **Figure 2B**, the pink test line appears at 10 min, and the line becomes clearer from 20 min to 35 min. There is no significant difference between the 20 min and 25 min lines. Therefore, a response time of 20 min was chosen for the RPA-LFS test of *C*. *tropicalis*. Optimal conditions for *C*. *tropicalis* RPA-LFS were determined to be incubation at 39°C for 20 min.

### Sensitivity Determination of the RPA-LFS Assay System

**Figure 3:**
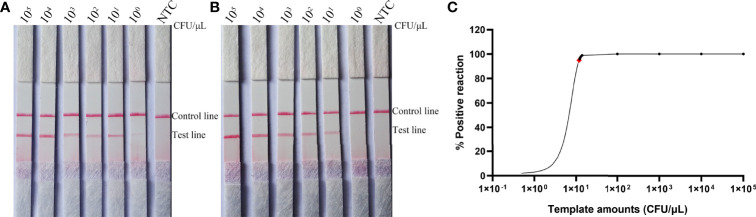
Determination of the lower limit of detection of the *C. tropicalis* RPA-LFS assay. **(A)** The lowest limit of detection for the *C. tropicalis* RPA-LFS assay system established using primer-probe set F5/P/R1B was determined from eight independent assays using inactivating solutions of *C. tropicalis* with serial dilutions from 10^0^ to 10^5^ CFU for each reaction. Image shows the results of the RPA-LFS with the number of templates indicated at the top of the bar graph. **(B)** Image of the results of the RPA-LFS assay using primer-probe set F5/P/R1B and 10^5^ CFU of *C. albicans* as interference. **(C)** Probit regression analysis was performed on data collected from eight replicates.

To determine the detection limit of the RPA-LFS system for the detection of *C*. *tropicalis*, 10-fold gradient dilutions of the *C*. *tropicalis* genome ranging from 10^0^ to 10^5^ CFU/µL were tested (reaction volume: 50 µL, 1 µL of *C*. *tropicalis* inactivation solution was added to each reaction). Although weak, a pink band still appeared on the test line at 10 CFU/µL. Moreover, the pink band became darker as the concentration of *C*. *tropicalis* increased (**Figure 3A**). To test whether the system is resistant to interference from other fungal DNA, 10^5^ CFU/µL of inactivating solution of another common pathogen, *C*. *albicans*, was added to the RPA reaction. The *C*. *albicans* inactivation solution did not interfere with the detection of *C*. *tropicalis* (**Figure 3B**). We conclude that the lower limit of detection of the RPA-LFS system is 10 CFU/50 µL per reaction in the presence of unpurified DNA and that this detection sensitivity is not affected by the presence of other fungi. In addition, not all assays produced positive results with 10^0^ (six positive results out of eight samples, 6/8) and 10 (1/8) CFU of the template. To further determine the lower limit of detection for the RPA-LFS assay, data from eight independent assays were statistically analyzed for probit regression analysis. With a 95% probability, the lowest lower limit of detection was 9.94 CFU per reaction (**Figure 3C**).

### Interspecies Specificity Determination of the RPA-LFS Assay System

**Figure 4:**
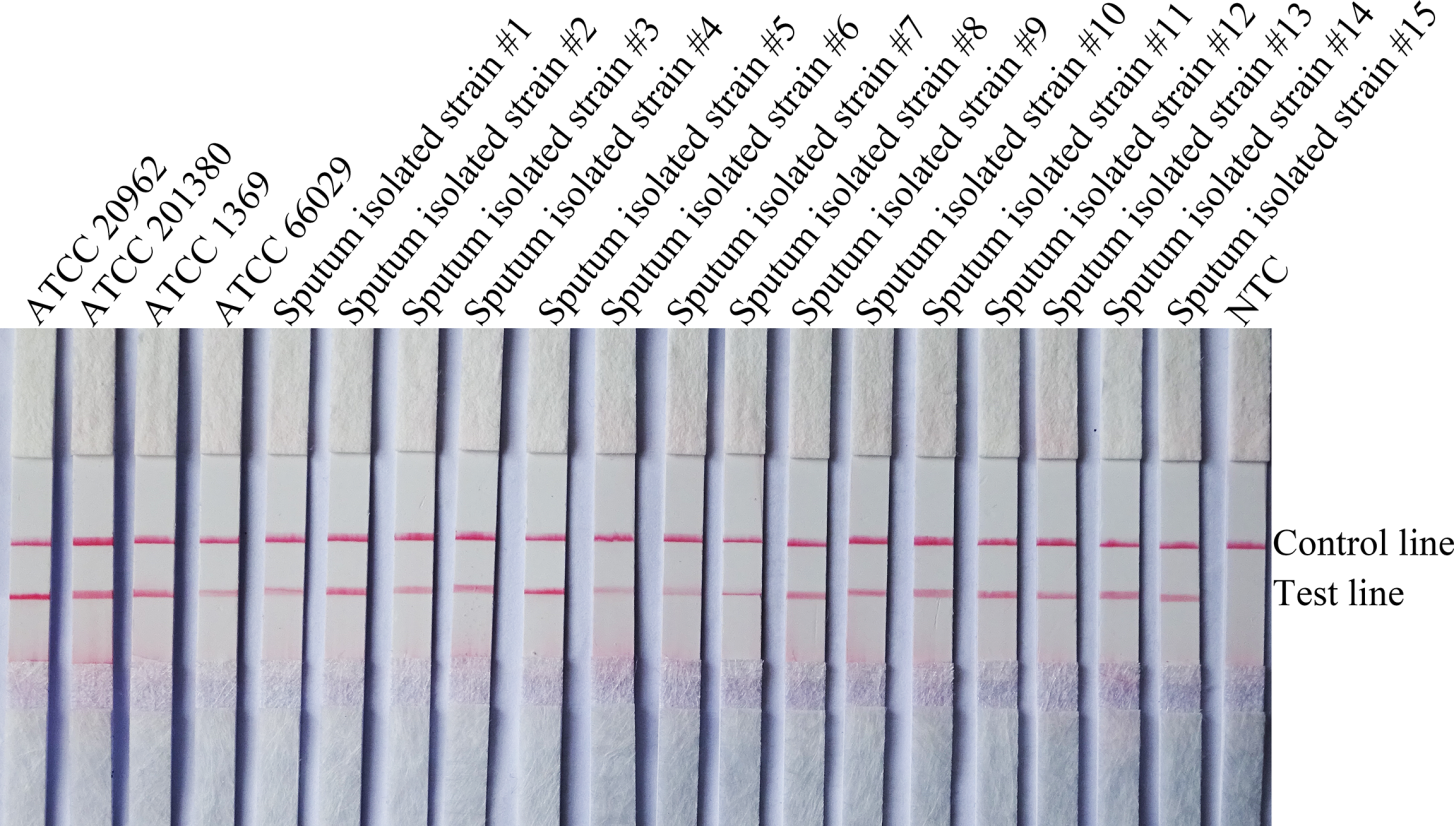
Validation of the specificity of primer pair F5/P/R1B against *C*. *tropicalis*. #1–15 refers to fifteen isolates of *C*. *tropicalis* from clinical samples, and NTC indicates no template control. The positions of the test and control lines are marked on the right side of the bar graph. Reactions were performed at 39°C for 20 min. Images are representative of three independent experiments.

**Figure 5:**
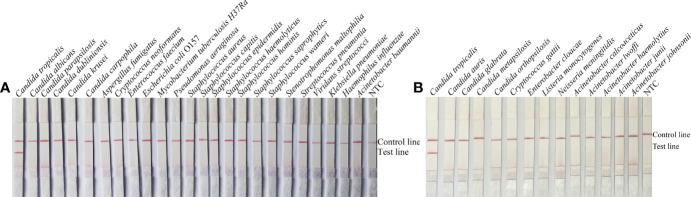
Specificity of F5/P/R1B. **(A, B)** *C*. *tropicalis* ATCC 20962 was used as a positive control against other pathogenic bacteria tested, and species names are indicated at the top of each strip. NTC indicates no template control. The positions of the test and control lines are marked on the right side of the bars. Reactions were performed at 39°C for 20 min. Images are representative of three independent experiments.

To confirm the inclusivity and specificity of F5/R1B/P, RPA-LFS amplification was performed on four reference strains, fifteen clinical isolates, and other pathogenic bacteria (**Table 2**). Four reference strains and fifteen clinical isolates showed positive results (**Figure 4**), while all other pathogenic cultures were negative (**Figures 5A, B**), indicating that the primer-probe set showed good inclusion and specificity for *C*. *tropicalis* without cross-reactivity with other pathogenic bacteria, and specificity without cross-reactivity with other pathogenic bacteria, indicating that the system can effectively and accurately detect *C*. *tropicalis*.

**Table 2:** Bacterial strains used in this study.

Species	Source	Strain designation
*Candida tropicalis*	Reference strain	ATCC 20962
*C. tropicalis*	Reference strain	ATCC 201380
*C. tropicalis*	Reference strain	ATCC 1369
*C. tropicalis*	Reference strain	ATCC 66029
*C. tropicalis*	Sputum isolated strain	#1 #2 #3 #4 #5 #6 #7 #8 #9 #10 #11 #12 #13 #14 #15
*Candida albicans*	Reference strain	ATCC 10231
*Candida parapsilosis*	Sputum isolated strain	N/A
*Candida dubliniensis*	Sputum isolated strain	N/A
*Candida krusei*	Sputum isolated strain	N/A
*Candida carpophila*	Sputum isolated strain	N/A
*Aspergillus fumigatus*	Sputum isolated strain	N/A
*Cryptococcus neoformans*	Reference strain	ATCC 14116
*Enterococcus faecium*	Sputum isolated strain	N/A
*Escherichia coli O157*	Sputum isolated strain	N/A
*Mycobacterium tuberculosis H37Ra*	Sputum isolated strain	N/A
*Pseudomonas aeruginosa*	Sputum isolated strain	N/A
*Staphylococcus aureus*	Sputum isolated strain	N/A
*Staphylococcus capitis*	Sputum isolated strain	N/A
*Staphylococcus epidermidis*	Sputum isolated strain	N/A
*Staphylococcus haemolyticus*	Sputum isolated strain	N/A
*Staphylococcus hominis*	Sputum isolated strain	N/A
*Staphylococcus saprophytics*	Sputum isolated strain	N/A
*Staphylococcus wameri*	Sputum isolated strain	N/A
*Stenotrophomonas maltophilia*	Sputum isolated strain	N/A
*Streptococcus pneumonia*	Sputum isolated strain	N/A
*Viridans streptococci*	Sputum isolated strain	N/A
*Klebsiella pneumoniae*	Sputum isolated strain	N/A
*Haemophilus influenzae*	Reference strain	ATCC 49247
*Acinetobacter baumannii*	Reference strain	ATCC 19606
*Candida auris*	Reference strain	N/A
*Candida glabrata*	Reference strain	N/A
*Candida metapsilosis*	Reference strain	N/A
*Candida orthopsilosis*	Reference strain	N/A
*Cryptococcus gattii*	Reference strain	N/A
*Enterobacter cloacae*	Reference strain	N/A
*Listeria monocytogenes*	Reference strain	N/A
*Neisseria meningitidis*	Reference strain	N/A
*Acinetobacter calcoaceticus*	Reference strain	N/A
*Acinetobacter lwoffi*	Reference strain	N/A
*Acinetobacter haemolytius*	Reference strain	N/A
*Acinetobacter junii*	Reference strain	N/A
*Acinetobacter johnsonii*	Reference strain	N/A

ATCC, American Type Culture Collection (Manassas, VA, USA).

### Clinical Sample Assay

To verify the practical application of RPA-LFS, 191 clinical samples were collected for RPA-LFS and qPCR assays, including 93 blood samples, 45 sputum samples, 36 urine samples, and 17 other samples. The results are shown in **Table 3**. A total of 67 samples belonged to *C*. *tropicalis*, and the detection rate was 35.08%. Positive tests more commonly found in blood, this is consistent with detection using qPCR and traditional culture methods.

**Table 3:** Prevalence of *ITS2* gene in 191 clinical samples of *C*. *tropicalis* using RPA-LFS and qPCR (summarized).

RPA-LFS assay				
		Positive	Negative	Total
qPCR	Positive	67	0	67 (blood 35, sputum 17, urine 12, and other samples 3)
	Negative	0	124	124 (blood 58, sputum 28, urine 24, and other samples 14)
Total		67	124	191

## Discussion

*C*. *tropicalis* is a typical conditional pathogenic yeast, and this opportunistic fungal pathogen is responsible for both mucosal and disseminated infection. Therefore, a rapid and sensitive diagnosis of *C*. *tropicalis* in the early stages of infection is important to ensure patient safety (Singh et al., 2020; Ardizzoni et al., 2021). Pathogen detection requires extraction of high-quality fungal genomic DNA. Some fungi are multinucleated and rich in secondary metabolites such as polysaccharides and proteins. Conventional traditional methods use liquid nitrogen grinding for cell wall disruption; liquid nitrogen combined with proteinase K and snailase enzymatic disruption of the cell wall is also used. Fungal nucleic acids are often tightly bound to proteins, and the key to extracting high-quality fungal DNA is to effectively separate nucleic acids from proteins. Most methods to disrupt the cell membrane to depolymerize nucleic acids and proteins use the chemical cetyl trimethyl ammonium bromide (CTAB), which not only depolymerizes nucleic acids and proteins but also removes complex metabolic products such as intracellular polysaccharides (Huang et al., 2018). For the removal of proteins released by cell rupture, phenol and mercaptoethanol are commonly used; these drugs very toxic and harmful to humans. These methods consume a large number of chemical reagents, include cumbersome experimental steps, long extraction time, and most importantly, more exposure to toxic reagents. Overall, they do not meet the current needs of high-throughput fungal genome research, so a one-step method for rapid release of genomic DNA is difficult for fungal detection. Drawing on the experience of bacterial genomic DNA extraction, boiling can partially release the genome of *C*. *tropicalis* for use in the RPA-LFS assay (Rosenbaum et al., 2019).

Molecular detection techniques require the selection of diagnostic amplification targets to effectively detect specific species. Several studies have evaluated various methods for detecting *C*. *tropicalis* infection in clinical samples from different sources. Among these methods, the *ITS2* gene was most frequently used as a detection target (Turenne et al., 1999; Talukdar et al., 2021). Therefore, we designed a pair of primer probes to detect *C*. *tropicalis*. The present study showed that there was no significant effect on the lower limit of detection for base mispairing and that RPA-LFS was able to accurately detect *C*. *tropicalis*. The minimum lower limit of detection for the RPA-LFS assay was 10 CFU, and this sensitivity was the same as that of the on-site RPA method, which is 10^0^–10 CFU per reaction. In addition, the RPA-LFS assay for detecting *C*. *tropicalis* is simple and rapid; the assay can be completed in less than 30 min (5 min for sample pre-treatment, 20 min for amplification, and 5 min for LFS analysis). This method requires only an isothermal temperature of 37°C, which can be achieved with body temperature heating. Following the relatively simple instructions, the assay results can be easily read without instrumentation. By contrast, PCR, qPCR, and LAMP methods require temperature-controlled equipment and relatively long reaction times. Real-time RPA methods require a shorter time than PCR but require the use of a fluorescence detector. The total cost of real-time RPA is higher than the cost of the RPA-LFS assay (Zeng et al., 2019). Evaluation of clinical samples showed that the accuracy of the RPA-LFS essay was good. Testing of samples from different patients showed that testing of positive samples with RPA-LFS was comparable to qPCR (Asadzadeh et al., 2019), indicating that the RPA-LFS assay presents an alternative assay. Compared with MALDI TOF, RPA-LFS allows for direct detection of suspected Candida infections without the need for lengthy pure culture time, and it has been reported that multiplex RPA-LFS is used for the detection of common pathogenic bacteria (Wang et al., 2021), indicating that the technique can have the ability to differentiate multiple species in a single assay as MALDI-TOF does. However, RPA-LFS has some limitations, as one strip can identify no more than three species simultaneously, but it can also be compensated by multiplexing multiplexed test strips with different markers and developing related detection devices to detect multiple pathogens simultaneously. In conclusion, the established RPA-LFS assay is simple, rapid, accurate, does not require laboratory facilities, and can be combined with a simple and rapid DNA extraction method for home detection of infections caused by *C*. *tropicalis*, and timely diagnosis can facilitate early treatment of this fungal infection.

## Conclusion

Recombinant enzyme polymerase isothermal amplification combined with colloidal gold test strips (RPA-LFS) developed in this study is a rapid molecular diagnostic technique for *C*. *tropicalis*; test results can be obtained in 30 min. This method has high specificity, high sensitivity, low instrument dependence, does not require trained laboratory personnel, is highly operable, and can be performed on-site to meet the needs of bedside diagnosis. It also meets the needs of remote hospitals with weak conditions. Overall, this assay will be important for rapid detection of *C*. *tropicalis*.

## Data Availability Statement

The original contributions presented in the study are included in the article/supplementary material. Further inquiries can be directed to the corresponding authors.

## Author Contributions

XG, YM, and LW designed the experiments and wrote the manuscript. CX, YW, and KW collected the clinical samples. FW, PZ, and LW performed the main experiments. WZ and LW analyzed the data. All authors contributed to the article and approved the submitted version.

## Funding

This study was supported by grants from the High-level Innovation and Entrepreneurship Talents Introduction Program of Jiangsu Province of China (grant number: 2019-30345), the “521 Project” scientific research funding project of Lianyungang City (grant number: LYG06521202157), the “HaiYan Plan” scientific research funding project of Lianyungang City (grant number: 2019-QD-008), the Clinical Medical Science and Technology Development Fund of Jiangsu University (grant number: JLY20180020). the China Agriculture Research System of MOF and MARA, National Key R&D Program of China, the key projects of International Scientific and Technological Innovation Cooperation (grant number: 2021YFE0111100), and the Zhenjiang Science and Technology Support Project (grant no. GJ2021015).

## Conflict of Interest

The authors declare that the research was conducted in the absence of any commercial or financial relationships that could be construed as a potential conflict of interest.

## Publisher’s Note

All claims expressed in this article are solely those of the authors and do not necessarily represent those of their affiliated organizations, or those of the publisher, the editors and the reviewers. Any product that may be evaluated in this article, or claim that may be made by its manufacturer, is not guaranteed or endorsed by the publisher.

## References

[B1] Ardizzoni A. Wheeler R. T. Pericolini E. (2021). It Takes Two to Tango: How a Dysregulation of the Innate Immunity, Coupled With Candida Virulence. Triggers VVC Onset 12, 692491. doi: 10.3389/fmicb.2021.692491 PMC8215348 34163460

[B2] Asadzadeh M. Alanazi A. F. Ahmad S. Al-Sweih N. Khan Z. (2019). Lack of Detection of Candida Nivariensis and Candida Bracarensis Among 440 Clinical Candida Glabrata Sensu Lato Isolates in Kuwait. PloS One 14 (10), e0223920. doi: 10.1371/journal.pone.0223920 31618264 PMC6795469

[B3] Baixench M. T. Taillandier A. Paugam A. (2006). Clinical and Experimental Evaluation of a New Chromogenic Medium (OCCA, Oxoid) for Direct Identification of Candida Albicans, C. Tropicalis and C. Krusei. Mycoses 49 (4), 311–315. doi: 10.1111/j.1439-0507.2006.01259.x 16784446

[B4] Cossio A. Jojoa J. Castro M. D. M. Castillo R. M. Osorio L. Shelite T. R.. (2021). Diagnostic Performance of a Recombinant Polymerase Amplification Test-Lateral Flow (RPA-LF) for Cutaneous Leishmaniasis in an Endemic Setting of Colombia. PloS Negl. Trop. Dis. 15 (4), e0009291. doi: 10.1371/journal.pntd.0009291 33909619 PMC8081229

[B5] Dai T. Yang X. Hu T. Jiao B. Xu Y. Zheng X.. (2019). Comparative Evaluation of a Novel Recombinase Polymerase Amplification-Lateral Flow Dipstick (RPA-LFD) Assay, LAMP, Conventional PCR, and Leaf-Disc Baiting Methods for Detection of Phytophthora Sojae. Front. Microbiol. 10. doi: 10.3389/fmicb.2019.01884 PMC6696978 31447827

[B6] Frimpong M. Ahor H. S. Wahed A. A. E. Agbavor B. Sarpong F. N. Laing K.. (2019). Rapid Detection of Mycobacterium Ulcerans With Isothermal Recombinase Polymerase Amplification Assay. PloS Negl. Trop. Dis. 13 (2), e0007155. doi: 10.1371/journal.pntd.0007155 30707706 PMC6373974

[B7] García-Salazar E. Acosta-Altamirano G. Betancourt-Cisneros P. Reyes-Montes M. D. R. Rosas-De-Paz E. Duarte-Escalante E.. (2022). Detection and Molecular Identification of Eight Candida Species in Clinical Samples by Simplex PCR. Microorganisms 10, (2). doi: 10.3390/microorganisms10020374 PMC8880469 35208828

[B8] Huang X. Duan N. Xu H. Xie T. N. Xue Y. R. Liu C. H. (2018). CTAB-PEG DNA Extraction From Fungi With High Contents of Polysaccharides. Mol. Biol. 52 (4), 621–628. doi: 10.1134/S0026893318040088 30113038

[B9] Hu J. Huang R. Sun Y. Wei X. Wang Y. Jiang C.. (2019). Sensitive and Rapid Visual Detection of Salmonella Typhimurium in Milk Based on Recombinase Polymerase Amplification With Lateral Flow Dipsticks. J. Microbiol. Methods 158, 25–32. doi: 10.1016/j.mimet.2019.01.018 30703446

[B10] Khodadadi H. Karimi L. Jalalizand N. Adin H. Mirhendi H. (2017). Utilization of Size Polymorphism in ITS1 and ITS2 Regions for Identification of Pathogenic Yeast Species. J. Med. Microbiol. 66 (2), 126–133. doi: 10.1099/jmm.0.000426 28260588

[B11] Liu X. Yan Q. Huang J. Chen J. Guo Z. Liu Z.. (2019). Influence of Design Probe and Sequence Mismatches on the Efficiency of Fluorescent RPA. World J. Microbiol. Biotechnol. 35 (6), 95. doi: 10.1007/s11274-019-2620-2 31187258

[B12] Makino H. Fujimoto J. Watanabe K. (2010). Development and Evaluation of a Real-Time Quantitative PCR Assay for Detection and Enumeration of Yeasts of Public Health Interest in Dairy Products. Int. J. Food Microbiol. 140 (1), 76–83. doi: 10.1016/j.ijfoodmicro.2010.02.004 20223545

[B13] Piepenburg O. Williams C. H. Stemple D. L. Armes N. A. (2006). DNA Detection Using Recombination Proteins. PloS Biol. 4 (7), 1115–1121. doi: 10.1371/journal.pbio.0040204 PMC1475771 16756388

[B14] Rosenbaum J. Usyk M. Chen Z. Zolnik C. P. Jones H. E. Waldron L.. (2019). Evaluation of Oral Cavity DNA Extraction Methods on Bacterial and Fungal Microbiota. Sci. Rep. 9 (1), 1531. doi: 10.1038/s41598-018-38049-6 30728424 PMC6365504

[B15] Rosser A. Rollinson D. Forrest M. Webster B. L. J. P. (2015). Isothermal Recombinase Polymerase Amplification (RPA) of Schistosoma Haematobium DNA and Oligochromatographic Lateral Flow Detection. Vectors 8, 446. doi: 10.1186/s13071-015-1055-3 PMC4559068 26338510

[B16] Singh D. K. Tóth R. Gácser A. (2020). Mechanisms of Pathogenic Candida Species to Evade the Host Complement Attack. Front. Cell. Infect. Microbiol. 10, 94. doi: 10.3389/fcimb.2020.00094 32232011 PMC7082757

[B17] Talukdar R. Padhi S. Rai A. K. Masi M. Evidente A. Jha D. K.. (2021). Isolation and Characterization of an Endophytic Fungus Colletotrichum Coccodes Producing Tyrosol From Houttuynia Cordata Thunb. Using ITS2 RNA Secondary Structure and Molecular Docking Study. Front. Bioeng. Biotechnol. 9, 650247. doi: 10.3389/fbioe.2021.650247 34222209 PMC8249321

[B18] Trabasso P. Matsuzawa T. Fagnani R. Muraosa Y. Tominaga K. Resende M. R.. (2015). Isolation and Drug Susceptibility of Candida Parapsilosis Sensu Lato and Other Species of C. Parapsilosis Complex From Patients With Blood Stream Infections and Proposal of a Novel LAMP Identification Method for the Species. Mycopathologia 179 (1), 53–62. doi: 10.1007/s11046-014-9830-9 25481844

[B19] Turenne C. Y. Sanche S. E. Hoban D. J. Karlowsky J. A. Kabani A. M. (1999). Rapid Identification of Fungi by Using the ITS2 Genetic Region and an Automated Fluorescent Capillary Electrophoresis System. J. Clin. Microbiol. 37 (6), 1846–1851. doi: 10.1128/jcm.37.6.1846-1851.1999 10325335 PMC84966

[B20] Wang F. Ge D. Wang L. Li N. Chen H. Zhang Z.. (2021). Rapid and Sensitive Recombinase Polymerase Amplification Combined With Lateral Flow Strips for Detecting Candida Albicans. Anal. Biochem. 633, 114428. doi: 10.1016/j.ab.2021.114428 34678249

[B21] Wang P. Liao L. Ma C. Zhang X. Yu J. Yi L.. (2021). Duplex On-Site Detection of Vibrio Cholerae and Vibrio Vulnificus by Recombinase Polymerase Amplification and Three-Segment Lateral Flow Strips. Biosens. (Basel) 11 (5), 151. doi: 10.3390/bios11050151 PMC8151630 34066017

[B22] Wang L. Wang Y. Wang F. Zhao M. Gao X. Chen H.. (2022). Development and Application of Rapid Clinical Visualization Molecular Diagnostic Technology for Cryptococcus Neoformans/C. Gattii Based on Recombinase Polymerase Amplification Combined With a Lateral Flow Strip. Front. Cell. Infect. Microbiol. 11, 803798. doi: 10.3389/fcimb.2021.803798 35096653 PMC8790172

[B23] Wu Y. D. Xu M. J. Wang Q. Q. Zhou C. X. Wang M. Zhu X. Q.. (2017). Recombinase Polymerase Amplification (RPA) Combined With Lateral Flow (LF) Strip for Detection of Toxoplasma Gondii in the Environment. Vet. Parasitol. 243, 199–203. doi: 10.1016/j.vetpar.2017.06.026 28807294

[B24] Yang H. Wang Y. Yang Q. Fan H. Wang L. Zhang T.. (2021). A Rapid and Sensitive Detection Method for Pseudomonas Aeruginosa Using Visualized Recombinase Polymerase Amplification and Lateral Flow Strip Technology. Front. Cell. Infect. Microbiol. 11, 698929. doi: 10.3389/fcimb.2021.698929 34595129 PMC8478171

[B25] Yan L. Zheng L. Zeng F. Y. Yang H. L. Wang Q. (2012). Study and Clinical Application of Real-Time PCR for Candida Tropicalis Infection Detection. J. Trop. Med. 12 (05), 550–52+580.

[B26] Yong P. V. C. Chong P. P. Lau L. Y. Yeoh R. S. C. Jamal F. (2008). Molecular Identification of Candida Orthopsilosis Isolated From Blood Culture. Mycopathologia 165 (2), 81–87. doi: 10.1007/s11046-007-9086-8 18266075

[B27] Zeng F. Wu M. Ma L. Han Z. Shi Y. Zhang Y.. (2019). Rapid and Sensitive Real-Time Recombinase Polymerase Amplification for Detection of Marek's Disease Virus. Mol. Cell. Probes 48, 101468. doi: 10.1016/j.mcp.2019.101468 31580913

[B28] Zhao L. de Hoog G. S. Cornelissen A. Lyu Q. Mou L. Liu T.. (2016). Prospective Evaluation of the Chromogenic Medium CandiSelect 4 for Differentiation and Presumptive Identification of Non-Candida Albicans Candida Species. Fungal Biol. 120 (2), 173–178. doi: 10.1016/j.funbio.2015.09.006 26781374

[B29] Zuza-Alves D. L. Silva-Rocha W. P. Chaves G. M. (2017). An Update on Candida Tropicalis Based on Basic and Clinical Approaches. Front. Microbiol. 8, 1927. doi: 10.3389/fmicb.2017.01927 29081766 PMC5645804

